# The “Most Wanted” Taxa from the Human Microbiome for Whole Genome Sequencing

**DOI:** 10.1371/journal.pone.0041294

**Published:** 2012-07-26

**Authors:** Anthony A. Fodor, Todd Z. DeSantis, Kristine M. Wylie, Jonathan H. Badger, Yuzhen Ye, Theresa Hepburn, Ping Hu, Erica Sodergren, Konstantinos Liolios, Heather Huot-Creasy, Bruce W. Birren, Ashlee M. Earl

**Affiliations:** 1 Department of Bioinformatics and Genomics, University of North Carolina at Charlotte, Charlotte, North Carolina, United States of America; 2 Bioinformatics Department, Second Genome, Inc., San Bruno, California, United States of America; 3 Department of Genetics, The Genome Institute, Washington University School of Medicine, St. Louis, Missouri, United States of America; 4 Microbial and Environmental Genomics Department. J. Craig Venter Institute, San Diego, California, United States of America; 5 School of Informatics and Computing, Indiana University, Bloomington, Indiana, United States of America; 6 Genome Sequencing and Analysis Program, Broad Institute of MIT and Harvard, Cambridge, Massachusetts, United States of America; 7 Earth Science Division, Ecology Department, Lawrence Berkeley National Laboratory, Berkeley, California, United States of America; 8 Microbial Genomics and Metagenomics Program, Department of Energy Joint Genome Institute, Walnut Creek, California, United States of America; 9 Institute for Genome Sciences, University of Maryland School of Medicine, Baltimore, Maryland, United States of America; University of Vienna, Austria

## Abstract

The goal of the Human Microbiome Project (HMP) is to generate a comprehensive catalog of human-associated microorganisms including reference genomes representing the most common species. Toward this goal, the HMP has characterized the microbial communities at 18 body habitats in a cohort of over 200 healthy volunteers using 16S rRNA gene (16S) sequencing and has generated nearly 1,000 reference genomes from human-associated microorganisms. To determine how well current reference genome collections capture the diversity observed among the healthy microbiome and to guide isolation and future sequencing of microbiome members, we compared the HMP’s 16S data sets to several reference 16S collections to create a ‘most wanted’ list of taxa for sequencing. Our analysis revealed that the diversity of commonly occurring taxa within the HMP cohort microbiome is relatively modest, few novel taxa are represented by these OTUs and many common taxa among HMP volunteers recur across different populations of healthy humans. Taken together, these results suggest that it should be possible to perform whole-genome sequencing on a large fraction of the human microbiome, including the ‘most wanted’, and that these sequences should serve to support microbiome studies across multiple cohorts. Also, in stark contrast to other taxa, the ‘most wanted’ organisms are poorly represented among culture collections suggesting that novel culture- and single-cell-based methods will be required to isolate these organisms for sequencing.

## Introduction

The human body is home to an enormous number and diversity of microbes. These microbes, the human microbiome, are increasingly thought to be required for normal human development, physiology, immunity, and nutrition [1]–[3]. While we owe many of these insights to 16S rRNA gene (16S)-based studies aimed at classifying and quantifying the microbes present among different people and body habitats [4]–[11], 16S sequences are insufficient proxies for the contents of the entire genome. Whole-genome sequences are an essential prerequisite for analyses that reveal how the metabolic potential of the microbiome might impact human health and disease. Moreover, a more complete set of assembled genomes from the human-associated microbiome will assist in the proper taxonomic and functional assignments of short sequence reads from whole-genome shotgun metagenomic, metatranscriptomic and metaproteomic studies, which are becoming increasingly feasible with the decreasing costs of DNA and protein sequencing methods.

The mission of the Human Microbiome Project (HMP) is to understand the role of human-associated microbial communities in health and disease. As part of this mission, the HMP seeks to generate a comprehensive reference collection of microbial genomes that represent the “healthy” human microbiome [12]–[14]. Through the efforts of the HMP and other sequencing projects, nearly 5,000 bacterial strains have been isolated from the human body, grown in culture and submitted for whole genome sequencing. While these organisms represent a wide range of taxonomic groups and origins of isolation, studies suggest that many microbes, including those that inhabit humans, have not been cultured and, thus, elude conventional methods for DNA preparation and sequencing [15], [16]. Consequently, reference genome collections remain incomplete. However, recent advances in culture- and single-cell-based technologies are making it possible to isolate and sequence these hard-to-culture microbes [17]–[19].

To guide these isolation efforts and selection of microbes from the microbiome for sequencing, we sought to identify and to create a list of high priority organisms from the human microbiome that remain un-represented in reference genome collections. This ‘most wanted’ list of organisms is meant to serve as a resource for the community interested in the isolation and sequencing of elusive members of the microbiome. To create the ‘most wanted’ list, we relied upon the HMP’s survey of healthy volunteers, the largest and most comprehensive survey of the human microbiome currently available [12], [14], [20], [21]. We compared the HMP’s 16S-based survey data from 18 different body habitats from more than 200 ‘healthy’ volunteers to other 16S reference collections. These comparisons helped us to define a scheme for prioritizing taxa that are both distantly related to already sequenced organisms and found frequently among the microbiome of HMP volunteers and other healthy humans.

The resulting ‘most wanted’ list of taxa is currently being used by the community for isolation and sequencing of previously un-sequenced organisms found in association with humans. The completion of these genomes will bring us closer to completing the reference genome collection and, hence, the gene-catalog of the human microbiome.

## Results

### A Modest Number of OTUs can Account for Nearly all of the Non-chimeric Sequences in the V1–V3 and V3–V5 HMP 16S Datasets

**Figure 1:**
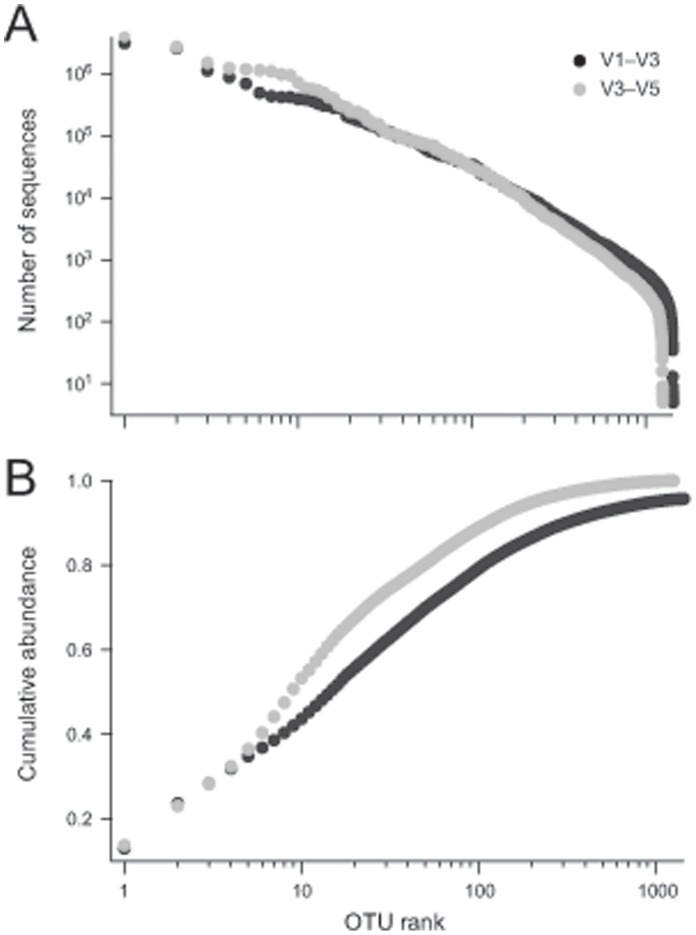
Rank Abundance curves for V1–V3 (black symbols) and V3–V5 (gray symbols) OTUs. (A) The number of sequences in each OTU. (B) Cumulative rank abundance. For both V1–V3 and V3–V5, on the order of 10–15 OTUs captured half of all individual sequences.

At the time of this study, the HMP’s survey of over 200 healthy volunteers and 18 body habitats was the single largest and most diverse data set from the human microbiome available [14]. As such, the HMP’s data were chosen to identify organisms from the human microbiome that had not yet been sequenced. To begin, we, separately, combined all available data from each of the HMP’s two major 16S-based surveys, targeting the V1–V3 and V3–V5 variable regions (Table 1). Each combined data set was clustered into operational taxonomic units (OTUs), using AbundantOTU [22] with the default setting of 97% average sequence identity (ID) for OTU inclusion. The resulting 1,440 V1–V3 and 1,258 V3–V5 OTUs contained nearly all (>95%) of the individual reads (Table 1). The majority of reads that did not cluster into OTUs were chimeric (as detected by UCHIME [23]) suggesting that many singleton reads represent PCR amplification error [24]. Rank abundance curves (Fig. 1) showed the unequal distribution of sequences within the OTUs; a small number of OTUs contained large numbers of 16S sequence reads while most OTUs contained many fewer reads. This pattern of a highly unequal distribution of sequences across OTUs is consistent with observations from other metagenomic surveys [25]–[27]. In addition, we observed a substantial number of chimeras within the HMP dataset, which were removed with the program UCHIME (Document S1 and Figures S1, S2, S3) [23], resulting in 773 V1–V3 and 695 V3–V5 non-chimeric HMP OTUs (hereafter referred to as the “HMP OTUs”). The sequence counts and average relative abundance for each chimeric and non-chimeric OTU, together with their consensus sequences, are available at http://hmpdacc.org/most_wanted.

**Table 1:** Number of samples and sequences included in the V1–V3 and V3–V5 analysis.

	V1–V3	V3–V5
Number of volunteers	180	239
Number of body sites	18	18
Total number of samples	3,321	5,061
Number of sequences	24,582,911	30,276,192
Sequences incorporated into an OTU	23,515,839 (95.6%)	29,567,447 (97.6%)
Percent of sequences not incorporated into OTUs that were chimeric(with UCHIME Gold as the reference DB)	61.7%	66.7%
Number of OTUs	1,440	1,258
Number of non-chimeric OTUs	773	695

### There were Few Novel and Many Uncultured Taxa within the HMP OTUs

**Figure 2:**
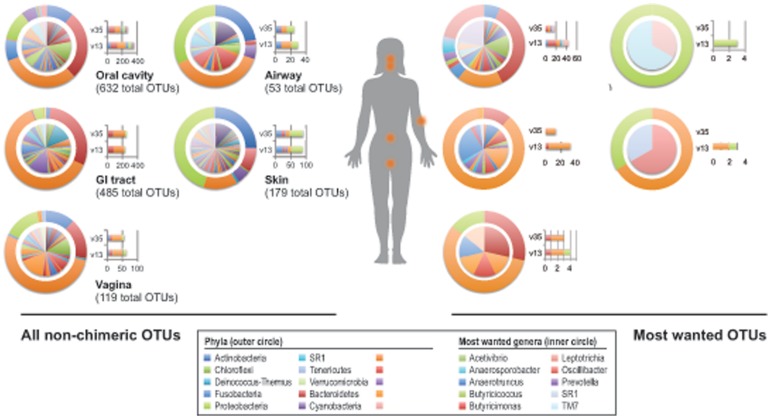
Body habitat distribution of non-chimeric and most wanted HMP OTUs. The distributions of 1,468 non-chimeric HMP OTUs (left panel) and 119 most wanted OTUs (right panel) are shown as phyla (outer circle) and genera (inner circle) at each of the 5 sampled body habitats. Distribution profiles were based on the habitat in which the HMP OTU was found most frequently. Bar graphs illustrate the relative proportion of HMP OTUs from each 16S variable region, shown as phyla. Color codes for all phyla and ‘most wanted’ genera with more than one representative are shown in left and right figure legends, respectively.

To understand which organisms were represented by the 773 V1–V3 and 695 V3–V5 non-chimeric HMP OTUs, we classified the consensus sequence associated with each HMP OTU using the RDP classifier (see Methods) [28]. As expected [29], we observed distinct phylogenetic communities in different body habitats (Fig. 2, left panel) reflecting the different microbial communities that inhabit the distinct niches within the human body. The observed community compositions were consistent across both V1–V3 and V3–V5 HMP OTUs, especially when phylum level classifications were taken into consideration (Fig. 2, left panel bar plots). However, even genus-level concordance was high; only ∼10% of HMP OTUs from each V region lacked a genus-level classification match to the other data set (data not shown).

**Figure 3:**
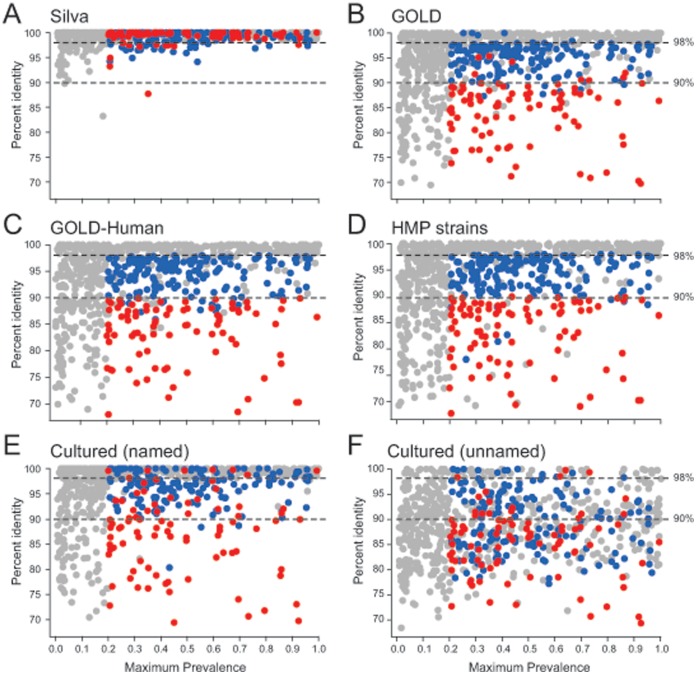
There were few novel, but many uncultured and unsequenced taxa within the HMP OTUs. Panels A through F present results from aligning HMP OTUs to six separate 16S sequence databases, indicated. For each HMP OTU, the y-axis of each panel shows the percent identity for the best matching sequence from the queried database, as determined by the program align.seqs in Mothur [30]. The x-axis of each panel shows the fraction of samples in which the OTU was present, at the body site of its highest prevalence. For example, a value of 0.5 means that the OTU was present in, at most, 50% of samples from a particular body site. The colors in all panels indicate assignment to priority groups for whole genome sequencing: red = highest priority, blue = medium priority, gray = low priority. Horizontal lines indicate 98% and 90% sequence identity.

To determine which HMP OTUs have not yet had a representative strain sequenced, we used the program align.seqs, within the package Mothur (see Methods) [30] to report the percent identity (across a global alignment) of the best matching sequence from several reference databases (Table S1) to each HMP OTU. We performed this search against v. 1.04 of the Silva database [31] (Fig. 3A), which is a comprehensive collection of full-length sequences as well as against numerous databases representing cultured or sequenced organisms (Fig. 3B–3F). When compared to the comprehensive Silva database (Fig. 3A), nearly all of the HMP OTUs had a compelling match (>98% identity) to a previously characterized full-length 16S sequence. From this, we concluded that there are only modest numbers of novel taxa within the HMP OTUs. We noted, however, that the taxonomic annotations given to the majority (70%) of matching Silva sequences did not include species designations and contained the word ‘uncultured’ or ‘clone’ indicating that these organisms may remain uncaptured. To directly determine which HMP OTUs represent taxa that have been cultured and sequenced, we also compared the HMP OTUs to (i) the GOLD database [32] (Fig. 3B), which represents microbes for which high-quality whole-genome sequences are available, (ii) the “GOLD-Human” database (Fig. 3C), which is a subset of the “GOLD” database representing strains isolated from the human body, (iii) “HMP strains” database (Fig. 3D), which represents whole-genome sequenced strains isolated from humans that are being completed as part of the HMP, (iv) the Greengenes “named” database (Fig 3E), which represents microbes (>3,000 genera and >7,000 species) that are in a culture collection and have been assigned a binomial name and (v) the Greengenes “unnamed” database (Fig. 3F), which represents microbes (5,869 16S sequences mostly from aquatic and terrestrial environments) that are in a culture collection, but lack binomial names. In all of these cases (Fig. 3B–3F), we see a similar pattern in which there are large numbers of HMP OTUs with poor matches to these reference collections. Taken together, these data demonstrate that while there are few truly novel 16S sequences in the HMP OTUs (Fig. 3A), there are many more taxa for which whole-genome sequences have not been determined (Fig. 3B–3D) and which have not been cultured (Fig. 3E-3F).

### Many HMP OTUs were Well Represented in Other Human Microbiome Cohorts

**Figure 4:**
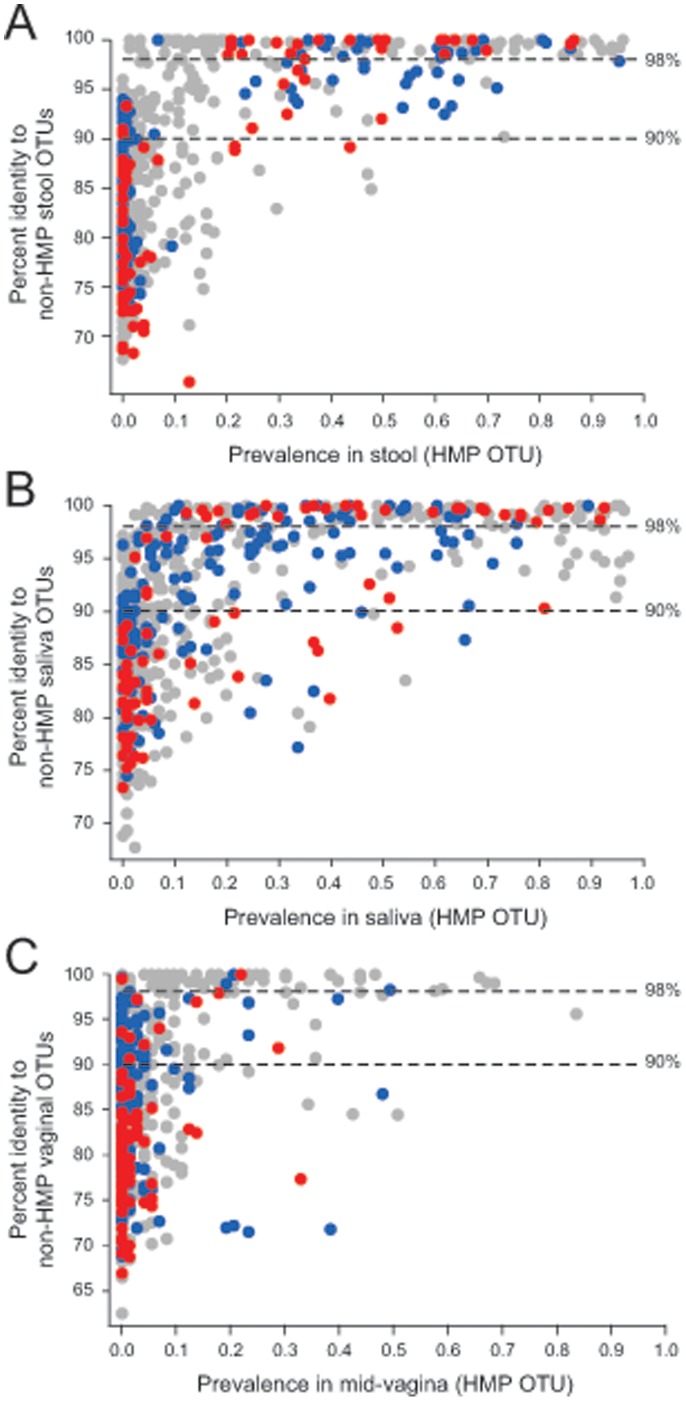
The most prevalent HMP OTUs were also present in other human cohorts. Databases were created from non-HMP enrolled healthy volunteers in which stool (4A), saliva (4B) and vaginal (4C) microbiomes were characterized (see Table S2). For each HMP OTU, the y-axis of each panel shows the percent identity for the best matching sequence from the queried database, as determined by the program align.seqs in Mothur [30]. The colors in all panels indicate assignment to priority groups for whole genome sequencing: red = highest priority, blue = medium priority, gray = low priority. Horizontal lines indicate 98% and 90% sequence identity.

While the close match of most of the HMP OTUs to the Silva database (Fig. 3A) indicated that most of the common taxa within the HMP OTUs have been seen before, the Silva database contained many sequences that are from environmental samples and may, therefore, not be relevant targets for the human microbiome. In order to determine whether the HMP OTUs are likely to be reproducibly observed in human cohorts, we compared the V1–V3 HMP OTUs to three recently completed metagenomic surveys of the human microbiome (details in Table S2) [33], [34]. Fig. 4A shows that the HMP OTUs most prevalent in stool were also largely present in the stool samples taken from the non-HMP cohort. We concluded from this that the more prevalent a taxa was within HMP stool samples, the more likely it is to be observed in other cohorts. A similar pattern was seen when comparing HMP V1–V3 saliva samples (Fig. 4B) and vaginal samples (Fig. 4C). We concluded that, at least for the most common taxa, there are sets of microbes that reproducibly appear in multiple cohorts. Since the organisms represented by these HMP OTUs were reproducibly observed across cohorts, obtaining their whole-genome sequences would represent a rich and universal resource that would inform multiple studies.

### Selection of a “Most Wanted” Group of Taxa Prioritized for Genome Sequencing

Though 97% 16S sequence identity is commonly used to define two organisms as the same ‘species’ and can perform well to distinguish species, there are many examples of how imperfectly 16S relatedness performs as a proxy for taxonomic and genomic relatedness [35], [36] including those that show significant genomic variation among organisms sharing >99% 16S sequence identity [37]. We, therefore, might posit that any HMP OTU lacking 100% identity to a sequenced genome has not been well represented among sequenced organisms and should be targeted for isolation and sequencing. While sequencing all organisms with less than perfect identity to an already sequenced organism would be ideal, we sought to define percent identity thresholds that would better help us to prioritize organisms for whole genome sequencing (described in Document S2 and Table S3).

We considered HMP OTUs that had less than 90% identity to either the GOLD-Human or HMP strains database to be “high priority” or “most wanted” taxa since these were most distant from already-sequenced genomes and are likely to represent un-sequenced genera (or higher level taxonomic groups *e.g.*, family) from the microbiome. We kept taxa in our “high priority” set even if they had a close match to the GOLD database because we reasoned that environmental strains, not associated with the human microbiota, might have significantly different genome contents even if the 16S rRNA genes were closely related. HMP OTUs with greater than 90% identity and less than 98% identity to GOLD-Human or HMP strains database were assigned to a “medium priority” group and are likely to represent un-sequenced species from the microbiome. Finally, HMP OTUs with greater than 98% identity to either the GOLD-Human or HMP strains database were put into a “low priority” group since these share the highest identity to already sequenced organisms isolated from humans. Also, because in this initial pass, we wanted to avoid directing resources toward isolating and sequencing microbes that are not prevalent within the human microbiome, we also assigned to the “low priority” group any microbe that did not occur in at least, 20% of samples from any body habitat. We reasoned that sets of taxa below this threshold might include many environmentally derived taxa that would not be reproducibly associated with the human microbiome. Supporting this, less frequent HMP OTUs were also less likely to share high identity to 16S data from other human derived samples (Fig. 4A–C) further suggesting that less frequent taxa are transient organisms from the microbiome, not held widely by healthy humans.

### Cultivated Organisms are Well Characterized by Whole-genome Sequencing

**Figure 5:**
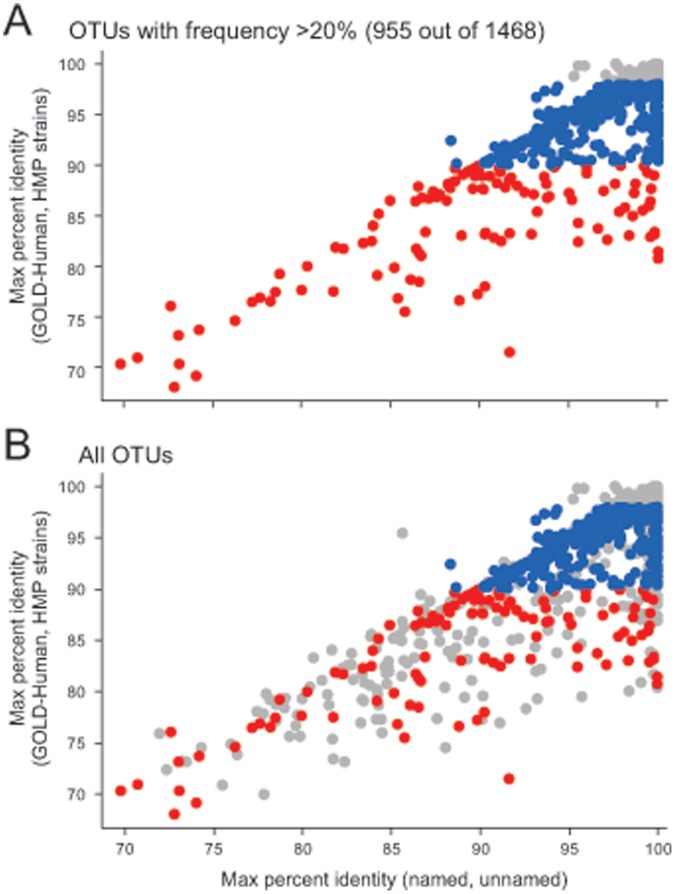
Nearly all sequenced taxa have been cultured but not all cultured taxa have been sequenced. For each taxa, the percent identity from the best match to a human sequenced database (GOLD-Human or HMP-strains) versus the best match to a sequence database of cultured organisms (named or unnamed). The colors in all panels indicate assignment to priority groups for whole genome sequencing: red = highest priority, blue = medium priority, gray = low priority. (A) OTUs that are present in at least 20% of all samples in at least one body habitat; (B) all HMP OTUs.

In order to determine to what extent taxa that have been sequenced are also the taxa that have been cultivated, we compared, for each HMP OTU consensus sequence, the best match within two 16S sequence databases of cultured organisms, “named” and “unnamed”, to the best match from a sequenced human database (GOLD Human or HMP strains) (Fig. 5). Almost without exception, taxa that have been whole-genome sequenced have an equal or better match in the databases of cultured organisms. Taxa within our “low priority” group were assigned that designation because they have >98% identity to a sequenced taxa; nearly every one of these taxa also had a >98% identity to a cultured taxon (Fig. 5A; grey symbols). This unsurprising result reflects current pipelines for microbial whole-genome sequencing that are dependent on culturing; while not every taxon with >98% identity to cultured organisms has been sequenced, nearly all sequenced taxa are present at high identity in the databases of cultured taxa. In contrast, only 30% of the 338 “medium priority” OTUs (blue symbols Figs. 3, 4, 5) and 17% of the 119 highest priority HMP OTUs, our “most wanted” (Figs. 3 4, 5, red symbols), had >98% identity to cultured organisms suggesting that completing the genome sequences for many of our “high” and “medium” priority organisms may require new methods for isolation. The fact that the “medium” and “most wanted” HMP OTUs were, on average, 10-fold less abundant than the low priority OTUs (average relative abundance was 0.015 versus 0.002) further suggests that it may take special culture- and, possibly, single cell-based methods to capture these less abundant, “most wanted” organisms.

### The “Most Wanted” Distribution and Hope for Capture

The right panel of Fig. 2 illustrates the relative distribution of the “most wanted”" HMP OTUs among the five major body habitats in which they were found most frequently. Though we did not attempt to identify “high priority” organisms from every body habitat, between 1.7% and 10% of total OTU diversity from body habitats (Fig. 2, left panel) were among our “most wanted” OTUs (Fig. 2, right panel). The taxonomic assignments of these “most wanted” were also diverse; 11 of 15 total bacterial phyla and 121 of 275 total bacterial genera identified within the HMP volunteers were represented. Though we observed that the proportion of V1–V3 and V3–V5 “most wanted” OTUs (Fig. 2, right panel bar plots) was not as consistent as for the total set of HMP OTUs (Figure 2, left panel bar plots), we were able to explain this by the identity threshold applied; V3–V5 HMP OTUs appeared to have less overall sequence divergence from our databases as compared to the V1–V3 HMP OTUs. We did not attempt to correct the V1–V3 and V3–V5 regions for different rates of evolution.

Finally, to demonstrate that the “most wanted” HMP OTUs can be captured, we compared HMP OTUs to publicly available 16S sequences from 238 bacterial single cells sorted from a stool sample taken from one healthy non-HMP volunteer (Roger Lasken, personal communication and http://hmpdacc.org/HMMDA16S/#data). Table S4 summarizes the global alignment results for this comparison. We identified 6 single cell 16S sequences with high identity to 6 different “most wanted” HMP OTUs belonging to the Firmicutes phylum (93.5–100% identity), all of which were found most frequently among stool samples of HMP volunteers. In addition, we identified 33 single cell 16S sequences with high identity to 12 HMP OTUs that met all of the criteria for “most wanted” inclusion except for their low (<20%) frequency among HMP stool samples. The presence of these 12 low frequency HMP OTUs, including one with identity to otu_1054_V1V3, previously identified as a novel organism related to *Barnesiella* [38], suggests that our frequency threshold of 20% might ultimately prove to be too strict and that some infrequent taxa might be present in the healthy human microbiome at a higher frequency than these data predict. Alternatively, the 16S sequencing depth achieved by the HMP with 454 technology may have been too low (with an average of 6,200 reads/sample) to reliably observe common, but low abundance taxa. Despite these concerns, these data demonstrate that the “most wanted” OTUs should be relatively easy to find. Certainly, our ability to identify and initiate whole-genome sequencing on more than 10% of all the “most wanted” OTUs from stool (Fig. 2, right panel) within a single stool sample supports the feasibility of our goal of constructing a comprehensive reference genome catalog of the human microbiome.

## Discussion

The goal of our project is to identify and create a prioritized list of common and unsequenced members of the microbiome for whole genome sequencing. We assert that a modest sequencing effort (on the order of one hundred “most wanted” taxa described in Table 2) combined with existing databases will result in genome sequences being available for a large majority of the most common microbial taxa present in the human microbiome. Generation of such a resource will assist in the ongoing efforts to understand how pathways encoded in microbial genomes contribute to human health and disease phenotypes and make more tractable phylogenetic assignment for short read whole-genome metagenomic experiments.

**Table 2:** The number of OTUs determined to be “high priority”, “medium priority” or “low priority” for full genome characterization.

	V1–V3	V3–V5	Both V regions
High Priority (Most Wanted)	85	34	119
Medium Priority	168	170	338
Low Priority	518	489	1011
TOTAL	773	695	1468

“Low priority” OTUs have a 98% identity to either GOLD-Human or HMP strains database or are seen in fewer than 20% of the samples from the body habitat in which they were observed most frequently. “Medium priority” OTUs had between a 90%–98% identity to either the GOLD or HMP strains database while “High priority” OTUs had less than a 90% identity to both the GOLD-Human or HMP database. (Both “Medium priority” and “High priority” OTUs, are present in at least 20% of the samples from the body habitat in which they were observed most frequently.).

We were able to achieve a simplified view of the human microbiome where a modest number of taxa (on the order of 1,000) were able to capture ∼95% of all the V1–V3 and V3–V5 HMP sequences (Fig. 1). The majority of the sequences not contained in an OTU were chimeric (Table 1) suggesting a high rate of error in unincorporated sequences. For this reason, we ignored all sequences not incorporated into an OTU. Removal of OTUs that had a chimeric consensus sequences (see Document S1) further simplified our view of the taxa present in the HMP OTUs with on the order of ∼800 non-chimeric OTUs found for both the V1–V3 and V3–V5 sequence sets (Table 1). These non-chimeric OTUs generally had a very close match in the Silva database (Fig. 3A). Because the Silva database largely reflects uncultured taxa, this is unsurprising. The most prevalent OTUs found in the HMP V1–V3 dataset were clearly also present in stool (Fig. 4A), saliva (Fig. 4B) and vaginal samples (Fig. 4C) from other cohorts. It was, therefore, clear that many of the same taxa occur across different subjects in multiple cohorts. While the 16S sequences representing these taxa were repeatedly observed across different experiment sets, many of these taxa have not yet been captured in culture collections (Fig. 3E-3F) or characterized with whole genome sequencing (Fig. 3B–3D).

The initial observations based on 454 pyrosequencing reported what appears to be near infinite diversity in environmental habitats [39], [40]. It currently remains unclear the degree to which such diversity reflects rare sequencing errors and chimerism. Because our study utilized the program AbundantOTU [22], which required construction of a consensus sequence from multiple reads in order to form an OTU, our analysis path deliberately avoided rare taxa. Our study is, therefore, neutral to the question of whether the rare biosphere represents true novel taxa or sequencing or PCR error. Moreover, our assignment of any taxa that was not seen in, at least, 20% of all samples from any body habitat to the “low priority” group further weights our priority lists against taxa that are not highly prevalent. We assert that emphasizing the sequencing of the most prevalent taxa first represents a rational deployment of sequencing resources. Of course, a limitation of this or any study that relies on the 16S rRNA view of a microbial community is that this one gene may not perfectly reflect the content of the rest of the genome or the evolutionary distance between organisms. Genome sequences will assist in this regard.

In this paper, we used percent identity from a global alignment as our metric to compare a query sequence to a reference database. Percent identity has some obvious advantages over other metrics. First, it is easy to calculate and makes intuitive sense, even to those without a background in phylogeny. Second, in a recent paper [41], it was shown that percent identity based on global alignments yielded more accurate matches to reference databases than a local alignment strategy based on best BLAST hit. There are, however, obvious disadvantages with the use of percent identity as a distance metric as well. Percent identity does not correct for different rates of evolution in different regions of the 16S sequence. This likely explains why we observed more “most wanted” V1–V3 OTUs than V3–V5 because the rate of evolution of V1–V3 is known to be more rapid [42]. An approach based on phylogenetic trees may have corrected for these sorts of differences by normalizing the background rate of evolution. We assert, however, that our collection of “most wanted” OTUs would be similar even if we had taken such an approach. When we used the phylogenetic tree-based method, pplacer [43], to place the HMP OTUs into a reference tree of sequenced taxa, we observed a highly overlapping set of HMP OTUs that were most distant from sequenced taxa on this tree and the “most wanted” taxa based on the global alignment criteria (data not shown). We are confident, therefore, that our results are not fundamentally a product of our choice of distance metric.

The HMP cohort was designed to measure the variation within healthy individuals. We would expect, therefore, that there will be some pathogenic taxa that are associated with disease that are not prevalent within the HMP cohort. We would anticipate future sequencing efforts to capture the genomes of these disease-associated microbes. As the cost of sequencing continues to decrease, and Illumina sequencing of 16S sequences becomes more common, the number of sequences per sample will increase well beyond the ∼6,000 sequences seen on average in HMP samples. In these future metagenomic sequencing experiments, some low abundance taxa that were not regularly detected with the sequencing depths of the 454-based HMP OTUs may appear as more highly prevalent. Nonetheless, given the current view of the human microbiome that is generated with the HMP OTUs through 454 sequencing technology, we assert that our list of high priority taxa is a reasonable use of resources to fill in the gaps of the phylogenetic tree representing the human microbiome.

The National Institutes of Health (NIH) is actively supporting the development of new culture- and single cell-based methods for bringing “most wanted” organisms to the sequencer. So far, the results have been very promising; the HMP is currently sequencing new isolates and single cells representing priority organisms. Though these efforts will continue, we appeal to the broader community to use the “most wanted” list, available at http://hmpdacc.org/most_wanted, to expand culture and genome collections to include these elusive members of the healthy microbiome. Finally, we believe that the simplified analysis path used to create the “most wanted” list can also be used to measure and direct progress of whole genome sequencing and culturing efforts for ongoing and future microbiome-related studies, human-related or otherwise.

## Methods

### Clustering 454 Data with AbundantOTU and Chimera Removal

V1–V3 and V3–V5 16S rRNA sequences were taken from the LQ HMP pipeline (from the files “hmp1.v13.lq.seq.summary” and “hmp1.v35.lq.seq.summary” provided in version 2.0 of the release to the 16S working group by Pat Schloss (ftp.hmpdacc.org;/16S/Production/Analysis/PPS-and-SRP002395-1.0/Schloss_Lab-2.0/finalData). The file “pds.metadata” within that release was used to assign subjects and body habitats. The non-HMP data sets, described in Table S2, were either downloaded from NCBI SRA (oral study, SRA024393, and vaginal study, SRP002463) or were obtained directly from the authors (stool study). The program AbundantOTU v2.0 and v4.2.40 [22], with the default parameters, was used to cluster 16S sequences from the HMP and non-HMP data, respectively (Table 1 and Table S2). Chimeric sequences were removed with the program UCHIME [23]. OTUs were considered chimeric if their consensus sequences were flagged by UCHIME in either de novo mode, in which the number of times each consensus sequence was observed was set to the number of reads which mapped to the corresponding OTU, or in the reference mode, where the reference was the GOLD database, which contains 16S sequences from fully sequenced genomes and therefore cannot contain chimeras. For non-HMP data sets, only the reference mode was used with GOLD serving as the database. The website, http://hmpdacc.org/most_wanted/, includes links to AbundantOTU output files that enable retrieval of the individual 454 reads ‘assigned’ to each HMP OTU.

### Global Alignment of OTUs

Consensus sequences for each OTU (provided in the cons output file from AbundantOTU available at http://hmpdacc.org/most_wanted/) were used to represent each taxa. Global alignments were performed against each reference database by using the program align.seqs in version 1.15 of Mothur [30]. For non-HMP data sets, version v1.20.3 of Mothur [30] was used to align HMP OTUs to non-HMP consensus sequences. For single cell analysis, version v1.20.3 of Mothur [30] was used to align single cell forward and reverse 16S sequences to a database of HMP consensus sequences. For each single cell, the 16S sequence with the highest aligning fraction (alignment length/query read length) was assessed for “most wanted” status. RDP taxonomy was assigned with version 2.1 of the standalone version of the RDP classifier [28]. No confidence criteria were enforced since selection of priority OTUs did not rely on accurate taxonomies. All of the databases for which the HMP OTUs were searched are listed in Tables S1 and S2 and the results of these searches are available at http://hmpdacc.org/most_wanted/.

### Creation of 16S Reference Data Sets

Greengenes [44], [45] holds publicly available 16S rRNA gene sequence records from NCBI >1250 bases in length and verified as 16S by NAST alignment [46]. Each reference data set was created as described in Table S1.

### Stool Single Cell Preparation and 16S Sequence Generation

Flow sorting and genomic DNA amplification from single microbial cells from fecal and oral samples was carried out according to Chitsaz *et. al.* [47] except a bacterial fraction was enriched from stool samples by nycodenz centrifugation [48] prior to sorting and both stool and oral cells were flow sorted into 2 µl of modified TE (10 mM Tris, 0.1 mM EDTA, pH8.0). Following cell lysis, MDAs were carried out using GenomiPhi HY Kit (GE Health Sciences) as per the manufacturer’s instructions except reactions were scaled to 12.5 µl volumes (personal communication from Roger S. Lasken). 16S amplification and sequencing was performed as described in Chitsaz et al. [47]. Stool single cell 16S sequences can be found at http://hmpdacc.org/HMMDA16S/#data by clicking the “Fecal Samples” link.

## Supporting Information

Figure S1 **The UCHIME ref score, against GOLD database, versus the fraction of reads chimeric in each OTU for each consensus sequence.** Left panel: V1–V3; right panel V3–V5. Gray indicates a consensus sequence called chimeric by UCHIME against the GOLD database.
(TIF)

Figure S2 **UCHIME Ref scores versus UCHIME de novo scores for V1–V3 (left panel) and V3–V5 (right panel).** Colors indicate whether the consensus sequence was called chimeric by just UCHIME de novo (red), just UCHIME ref to the GOLD database (gray), both methods (blue) or neither method (black).
(TIF)

Figure S3 **Silva percent identity versus max UCHIME score (max of UCHIME ref and UCHIME de novo) for V1–V3 (left panel) and V3–V5 (right panel).** Colors indicate whether the consensus sequence was called chimeric by just UCHIME de novo (red), just UCHIME ref to the GOLD database (gray), both methods (blue) or neither method (black).
(TIF)

Table S1 **Reference 16S sequence databases against which HMP OTUs were compared.**
(DOCX)

Table S2 **Non-HMP data sets against which HMP OTUs were compared.**
(DOCX)

Table S3 **Comparing species- (A) and genus-level (B) assignments to define percent identity cut-off values for prioritizing HMP OTUs. (See Document S2).**
(DOCX)

Table S4 **Comparison of single cell and HMP OTU consensus 16S sequences to identify “most wanted” single cells for whole genome sequencing.**
(DOCX)

Document S1 **Exploring high chimera rates among HMP OTUs.**
(DOCX)

Document S2 **Determining percent identity prioritization thresholds.**
(DOCX)
