# Supplementary figures and images for: The “Most Wanted” Taxa from the Human Microbiome for Whole Genome Sequencing
Source: PLoS One. 2012 Jul 26;7(7):e41294. doi: 10.1371/journal.pone.0041294 (PMC3406062; doi:10.1371/journal.pone.0041294)

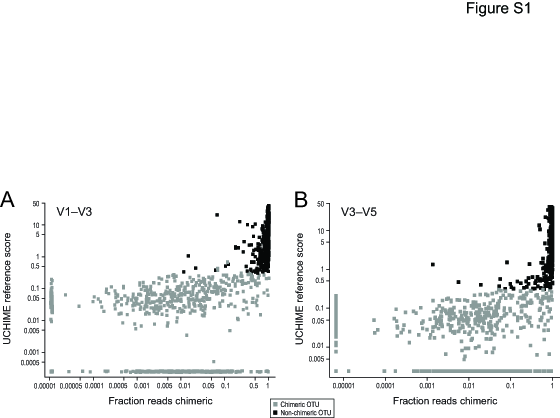

Supplement: Figure S1 — The UCHIME ref score, against GOLD database, versus the fraction of reads chimeric in each OTU for each consensus sequence. Left panel: V1–V3; right panel V3–V5. Gray indicates a consensus sequence called chimeric by UCHIME against the GOLD database. (TIF) [file pone.0041294.s001.tif]

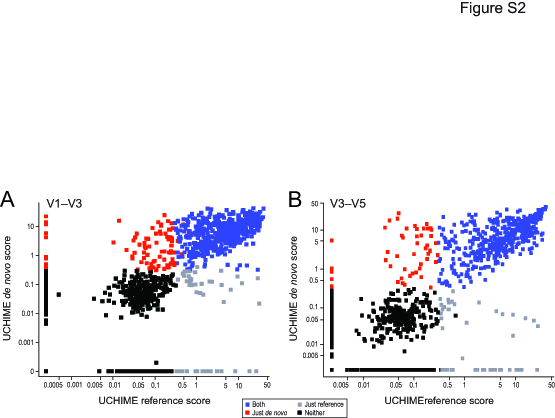

Supplement: Figure S2 — UCHIME Ref scores versus UCHIME de novo scores for V1–V3 (left panel) and V3–V5 (right panel). Colors indicate whether the consensus sequence was called chimeric by just UCHIME de novo (red), just UCHIME ref to the GOLD database (gray), both methods (blue) or neither method (black). (TIF) [file pone.0041294.s002.tif]

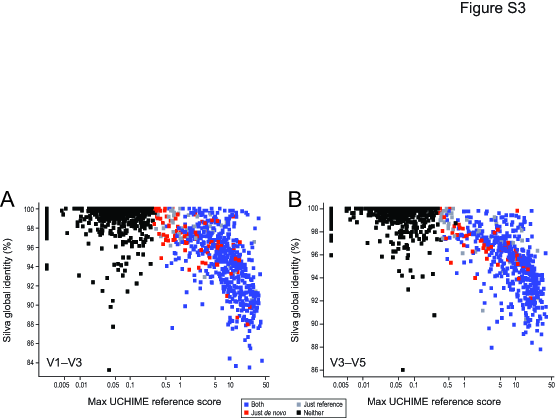

Supplement: Figure S3 — Silva percent identity versus max UCHIME score (max of UCHIME ref and UCHIME de novo) for V1–V3 (left panel) and V3–V5 (right panel). Colors indicate whether the consensus sequence was called chimeric by just UCHIME de novo (red), just UCHIME ref to the GOLD database (gray), both methods (blue) or neither method (black). (TIF) [file pone.0041294.s003.tif]
