# Supplementary material for: The “Most Wanted” Taxa from the Human Microbiome for Whole Genome Sequencing
Source: PLoS One. 2012 Jul 26;7(7):e41294. doi: 10.1371/journal.pone.0041294 (PMC3406062; doi:10.1371/journal.pone.0041294)
Supplement: Table S1 — Reference 16S sequence databases against which HMP OTUs were compared. (DOCX) [file pone.0041294.s004.docx]

**Table S1. The databases against which the HMP OTUs were compared.**

| **Comparative Data Sets** | **# sequences** | **Notes on creation** | **Download date** |
| --- | --- | --- | --- |
| **Silva- SSU_REF v. 104** | 512,037 | [31] |  |
| **GOLD** | 5,441 | Greengenes records where the gg ‘strain’ contains the GOLD ‘ORGANISM_NAME’ or the gg ncbi_tax_id equals the GOLD ‘TAXON_ID’ | **April 10, 2011** |
| **GOLD- Human** | 2,839 | Greengenes records in the GOLD subset where the GOLD ‘HOST NAME' is “Homo sapiens” | **May 11, 2011** |
| **HMP Strains** | 1,898 | Greengenes records in the GOLD subset where the GOLD ‘IMG_HMP_ID’ is registered. In cases where a strain’s 16S sequence was not found within a contig of the genome project, a sequence from the same species (else the same genus, else the same family) was substituted as surrogate. | **May 11, 2011** |
| **Isolated named strains** | 117,101 | Greengenes records derived from whole genome sequencing projects or records that contain binomial names in the genbank field ‘description’ and do not contain words such as “unnamed”, “unidentified” or “clone” in the genbank fields ‘description’, ‘source’ nor ‘title’. | **June 16, 2011** |
| **Isolated unnamed strains** | 5,869 | Greengenes records not in Named isolates and without binomial names in the genbank field ‘description’ and with genbank field ‘strain’ containing a designation. | **June 16, 2011** |
