# Supplementary material for: The “Most Wanted” Taxa from the Human Microbiome for Whole Genome Sequencing
Source: PLoS One. 2012 Jul 26;7(7):e41294. doi: 10.1371/journal.pone.0041294 (PMC3406062; doi:10.1371/journal.pone.0041294)
Supplement: Table S2 — Non-HMP data sets against which HMP OTUs were compared. (DOCX) [file pone.0041294.s005.docx]

**Table S2. Non-HMP data sets against which the HMP OTUs were compared.**

|  | # volunteers sampled | specific body habitat | # Raw Samples | Average Raw Read Depth per Sample | # AbundantOTUs | citation |
| --- | --- | --- | --- | --- | --- | --- |
| non-HMP Oral Study | 4 | saliva | 20 | 27360 | 230 | [34] |
| non-HMP Stool Study | 18 | stool | 107 | 12,092 | 457 | [33] |
| non-HMP Vaginal Study | 32 | mid-vagina & vaginal “smears” | 1173 | 2578 | 317 | (personal communication, Jacques Ravel) |
