# Supplementary material for: The “Most Wanted” Taxa from the Human Microbiome for Whole Genome Sequencing
Source: PLoS One. 2012 Jul 26;7(7):e41294. doi: 10.1371/journal.pone.0041294 (PMC3406062; doi:10.1371/journal.pone.0041294)
Supplement: Table S3 — Comparing species- (A) and genus-level (B) assignments to define percent identity cut-off values for prioritizing HMP OTUs. (See Document S2). (DOCX) [file pone.0041294.s006.docx]

**Table S3. Comparing species- and genus-level (B) assignments to define percent identity cut-off values for prioritizing HMP OTUs.** Comparison of genus- and species- level taxonomic assignments given to cultured organisms [‘named’ database] that share 100% 16S sequence identity to HMP OTUs to the genus- and species-level taxonomic assignment given to the HMP OTU’s best match from GOLD database.

| GOLD %identity range to HMP OTU | Total # names compared | # matching taxonomic assignments | # non-matching taxonomic assignments | % matching taxonomic assignment |
| --- | --- | --- | --- | --- |
| Species level comparisons | | | | |
| 100 | 103 | 97 | 6 | 94 |
| 99-99.999 | 34 | 32 | 2 | 94 |
| 98-98.999 | 4 | 4 | 0 | 100 |
| 97-97.999 | 10 | 3 | 7 | 30 |
| 96-96.999 | 11 | 0 | 11 | 0 |
| 95-95.999 | 6 | 0 | 6 | 0 |
| 90-94.999 | 14 | 0 | 14 | 0 |
| 83-89.999 | 6 | 0 | 6 | 0 |
| Genus level comparisons (OTUs with *sp.* designations excluded) | | | | |
| 100 | 126 | 119 | 7 | 94 |
| 99-99.999 | 47 | 46 | 1 | 98 |
| 98-98.999 | 6 | 6 | 0 | 100 |
| 97-97.999 | 17 | 13 | 4 | 76. |
| 96-96.999 | 19 | 15 | 4 | 79 |
| 95-95.999 | 9 | 6 | 3 | 67 |
| 90-94.999 | 24 | 17 | 7 | 71 |
| 83-89.999 | 14 | 8 | 6 | 57 |
