# Supplementary material for: The “Most Wanted” Taxa from the Human Microbiome for Whole Genome Sequencing
Source: PLoS One. 2012 Jul 26;7(7):e41294. doi: 10.1371/journal.pone.0041294 (PMC3406062; doi:10.1371/journal.pone.0041294)
Supplement: Table S4 — Comparison of single cell and HMP OTU consensus 16S sequences to identify “most wanted” single cells for whole genome sequencing. (DOCX) [file pone.0041294.s007.docx]

**Table S4. Comparison of single cell and HMP OTU consensus 16S sequences to identify ‘most wanted’ single cells for whole genome sequencing.** Table reports global alignment results from comparison of single cell and HMP OTU consensus sequences. Included are the characteristics of the best matching HMP OTU for each single cell sequence, including its priority status for sequencing. Bolded rows represent single cells with identity to HMP OTUs that met all of the criteria for ‘most wanted’ inclusion except for their low (<20%) frequency among HMP stool samples.

| **Single Cell Unique ID** | **Best Matching HMP OTU** | **Single Cell Global Identity to HMP OTU** | **HMP OTU Priority** | **HMP OTU Global Identity to GOLDhuman** | **HMP OTU Global Identity to HMP** | **HMP OTU Max Prevalence Body Habitat** | **HMP OTU Max Prevalence** |
| --- | --- | --- | --- | --- | --- | --- | --- |
| WGACA01T1F15187 | otu_600_V1V3 | 100 | HIGH /MOST WANTED | 82.2 | 82.2 | Stool | 0.67 |
| WGACA01T1R15117 | otu_203_V3V5 | 96.75 | HIGH /MOST WANTED | 88.13 | 88.13 | Stool | 0.33 |
| WGACA48030 | otu_430_V1V3 | 100 | HIGH /MOST WANTED | 79.78 | 79.78 | Stool | 0.61 |
| WGACA47909 | otu_158_V1V3 | 93.51 | HIGH /MOST WANTED | 87.29 | 87.29 | Stool | 0.7 |
| WGACA51481 | otu_171_V3V5 | 94.17 | HIGH /MOST WANTED | 83.69 | 83.69 | Stool | 0.36 |
| WGACA46T1F43561 | otu_998_V1V3 | 97.51 | HIGH /MOST WANTED | 76.71 | 78.42 | Stool | 0.2 |
| WGACA01T1F15082 | otu_568_V3V5 | 57.14 | MEDIUM | 93.16 | 93.19 | Subgingival plaque | 0.49 |
| WGACA01T1R15071 | otu_1082_V3V5 | 92.86 | MEDIUM | 96.46 | 96.46 | Stool | 0.29 |
| WGACA01T1R15273 | otu_89_V3V5 | 62.5 | MEDIUM | 93.37 | 93.37 | Anterior nares | 0.81 |
| WGACA01T1F40032 | otu_757_V3V5 | 97.34 | MEDIUM | 96.58 | 97.15 | Stool | 0.44 |
| WGACA01T1F40222 | otu_757_V3V5 | 96.77 | MEDIUM | 96.58 | 97.15 | Stool | 0.44 |
| WGACA01T1F40044 | otu_757_V3V5 | 97.34 | MEDIUM | 96.58 | 97.15 | Stool | 0.44 |
| WGACA01T1F40144 | otu_757_V3V5 | 96.97 | MEDIUM | 96.58 | 97.15 | Stool | 0.44 |
| WGACA01T1F40154 | otu_757_V3V5 | 97.15 | MEDIUM | 96.58 | 97.15 | Stool | 0.44 |
| WGACA39T1F39970 | otu_561_V3V5 | 99.75 | MEDIUM | 95.21 | 96.35 | Stool | 0.54 |
| WGACA01T1F40066 | otu_1151_V3V5 | 97.4 | MEDIUM | 97.96 | 97.96 | Stool | 0.27 |
| WGACA01T1F40167 | otu_757_V3V5 | 97.11 | MEDIUM | 96.58 | 97.15 | Stool | 0.44 |
| WGACA01T1F40276 | otu_757_V3V5 | 97.15 | MEDIUM | 96.58 | 97.15 | Stool | 0.44 |
| WGACA01T1F40189 | otu_757_V3V5 | 97.69 | MEDIUM | 96.58 | 97.15 | Stool | 0.44 |
| WGACA01T1F40308 | otu_298_V1V3 | 98.22 | MEDIUM | 96.34 | 97.26 | Stool | 0.86 |
| WGACA01T1F40446 | otu_757_V3V5 | 97.34 | MEDIUM | 96.58 | 97.15 | Stool | 0.44 |
| WGACA01T1F40353 | otu_561_V3V5 | 99.81 | MEDIUM | 95.21 | 96.35 | Stool | 0.54 |
| WGACA01T1F40642 | otu_899_V3V5 | 96.28 | MEDIUM | 95.69 | 96.08 | Stool | 0.4 |
| WGACA01T1F40460 | otu_757_V3V5 | 95.64 | MEDIUM | 96.58 | 97.15 | Stool | 0.44 |
| WGACA01T1F40654 | otu_561_V3V5 | 96.93 | MEDIUM | 95.21 | 96.35 | Stool | 0.54 |
| WGACA01T1F40686 | otu_757_V3V5 | 97.34 | MEDIUM | 96.58 | 97.15 | Stool | 0.44 |
| WGACA01T1F40406 | otu_757_V3V5 | 96.77 | MEDIUM | 96.58 | 97.15 | Stool | 0.44 |
| WGACA01T1F40502 | otu_757_V3V5 | 95.4 | MEDIUM | 96.58 | 97.15 | Stool | 0.44 |
| WGACA01T1R40346 | otu_249_V3V5 | 98.39 | MEDIUM | 97.01 | 97.26 | Stool | 0.83 |
| WGACA01T1R40554 | otu_492_V3V5 | 100 | MEDIUM | 93.76 | 93.76 | Stool | 0.51 |
| WGACA01T1R40384 | otu_561_V3V5 | 99.35 | MEDIUM | 95.21 | 96.35 | Stool | 0.54 |
| WGACA48041 | otu_854_V3V5 | 93.64 | MEDIUM | 97.12 | 97.12 | Stool | 0.48 |
| WGACA47946 | otu_854_V3V5 | 93.26 | MEDIUM | 97.12 | 97.12 | Stool | 0.48 |
| WGACA48066 | otu_854_V3V5 | 93.31 | MEDIUM | 97.12 | 97.12 | Stool | 0.48 |
| WGACA47897 | otu_633_V1V3 | 90.8 | MEDIUM | 95.97 | 95.97 | Stool | 0.55 |
| WGACA47898 | otu_633_V1V3 | 94.21 | MEDIUM | 95.97 | 95.97 | Stool | 0.55 |
| WGACA47806 | otu_757_V3V5 | 97.05 | MEDIUM | 96.58 | 97.15 | Stool | 0.44 |
| WGACA47826 | otu_854_V3V5 | 92.33 | MEDIUM | 97.12 | 97.12 | Stool | 0.48 |
| WGACA51204 | otu_561_V3V5 | 100 | MEDIUM | 95.21 | 96.35 | Stool | 0.54 |
| WGACA51210 | otu_324_V3V5 | 98.73 | MEDIUM | 96.2 | 96.46 | Stool | 0.75 |
| WGACA50865 | otu_757_V3V5 | 96.78 | MEDIUM | 96.58 | 97.15 | Stool | 0.44 |
| WGACA50874 | otu_298_V1V3 | 98.22 | MEDIUM | 96.34 | 97.26 | Stool | 0.86 |
| WGACA50881 | otu_757_V3V5 | 96.57 | MEDIUM | 96.58 | 97.15 | Stool | 0.44 |
| WGACA50919 | otu_298_V1V3 | 96.76 | MEDIUM | 96.34 | 97.26 | Stool | 0.86 |
| WGACA50938 | otu_298_V1V3 | 96.99 | MEDIUM | 96.34 | 97.26 | Stool | 0.86 |
| WGACA50983 | otu_757_V3V5 | 97.39 | MEDIUM | 96.58 | 97.15 | Stool | 0.44 |
| WGACA50991 | otu_757_V3V5 | 97.15 | MEDIUM | 96.58 | 97.15 | Stool | 0.44 |
| WGACA51006 | otu_298_V1V3 | 98.96 | MEDIUM | 96.34 | 97.26 | Stool | 0.86 |
| WGACA51020 | otu_298_V1V3 | 97.94 | MEDIUM | 96.34 | 97.26 | Stool | 0.86 |
| WGACA51038 | otu_298_V1V3 | 98.52 | MEDIUM | 96.34 | 97.26 | Stool | 0.86 |
| WGACA51044 | otu_757_V3V5 | 97.66 | MEDIUM | 96.58 | 97.15 | Stool | 0.44 |
| WGACA51048 | otu_757_V3V5 | 97.25 | MEDIUM | 96.58 | 97.15 | Stool | 0.44 |
| WGACA51056 | otu_899_V3V5 | 96.84 | MEDIUM | 95.69 | 96.08 | Stool | 0.4 |
| WGACA51057 | otu_561_V3V5 | 100 | MEDIUM | 95.21 | 96.35 | Stool | 0.54 |
| WGACA51107 | otu_757_V3V5 | 96.59 | MEDIUM | 96.58 | 97.15 | Stool | 0.44 |
| WGACA51126 | otu_899_V3V5 | 97.42 | MEDIUM | 95.69 | 96.08 | Stool | 0.4 |
| WGACA51159 | otu_757_V3V5 | 97.13 | MEDIUM | 96.58 | 97.15 | Stool | 0.44 |
| WGACA51160 | otu_561_V3V5 | 99.77 | MEDIUM | 95.21 | 96.35 | Stool | 0.54 |
| WGACA51188 | otu_757_V3V5 | 97.23 | MEDIUM | 96.58 | 97.15 | Stool | 0.44 |
| WGACA51220 | otu_1153_V3V5 | 88.74 | MEDIUM | 94.11 | 94.85 | Stool | 0.29 |
| WGACA51222 | otu_899_V3V5 | 98.63 | MEDIUM | 95.69 | 96.08 | Stool | 0.4 |
| WGACA51227 | otu_757_V3V5 | 97.67 | MEDIUM | 96.58 | 97.15 | Stool | 0.44 |
| WGACA51228 | otu_757_V3V5 | 96.96 | MEDIUM | 96.58 | 97.15 | Stool | 0.44 |
| WGACA51241 | otu_757_V3V5 | 97.15 | MEDIUM | 96.58 | 97.15 | Stool | 0.44 |
| WGACA51429 | otu_223_V3V5 | 98.91 | MEDIUM | 93.63 | 96.29 | Stool | 0.7 |
| WGACA51356 | otu_417_V1V3 | 100 | MEDIUM | 94.37 | 93.75 | Stool | 0.34 |
| WGACA51442 | otu_805_V3V5 | 92.95 | MEDIUM | 93.32 | 93.32 | Stool | 0.32 |
| WGACA51488 | otu_805_V3V5 | 91.3 | MEDIUM | 93.32 | 93.32 | Stool | 0.32 |
| WGACA51613 | otu_869_V3V5 | 98.85 | MEDIUM | 95.96 | 95.96 | Stool | 0.33 |
| WGACA51877 | otu_381_V3V5 | 99.8 | MEDIUM | 92.89 | 94.32 | Stool | 0.76 |
| WGACA41T1F43556 | otu_298_V1V3 | 95.91 | MEDIUM | 96.34 | 97.26 | Stool | 0.86 |
| WGACA42T1F43557 | otu_269_V1V3 | 99.71 | MEDIUM | 96.68 | 96.68 | Stool | 0.81 |
| WGACA01T1F43844 | otu_298_V1V3 | 97.12 | MEDIUM | 96.34 | 97.26 | Stool | 0.86 |
| WGACA01T1F43661 | otu_298_V1V3 | 97.12 | MEDIUM | 96.34 | 97.26 | Stool | 0.86 |
| **WGACA01T1F15058** | **otu_647_V1V3** | **94.32** | **LOW** | **78.47** | **79.5** | **Stool** | **0.19** |
| **WGACA01T1F15250** | **otu_1233_V3V5** | **92.72** | **LOW** | **84.6** | **84.6** | **Stool** | **0.07** |
| **WGACA01T1F15069** | **otu_647_V1V3** | **94.22** | **LOW** | **78.47** | **79.5** | **Stool** | **0.19** |
| **WGACA01T1F15189** | **otu_647_V1V3** | **95.15** | **LOW** | **78.47** | **79.5** | **Stool** | **0.19** |
| **WGACA01T1F15130** | **otu_1233_V3V5** | **92.21** | **LOW** | **84.6** | **84.6** | **Stool** | **0.07** |
| **WGACA01T1F15136** | **otu_1233_V3V5** | **97.19** | **LOW** | **84.6** | **84.6** | **Stool** | **0.07** |
| **WGACA01T1F15137** | **otu_666_V1V3** | **93.17** | **LOW** | **77.34** | **77.34** | **Stool** | **0.15** |
| **WGACA01T1F15226** | **otu_774_V3V5** | **93.12** | **LOW** | **85.79** | **85.98** | **Stool** | **0.14** |
| **WGACA01T1F15227** | **otu_1233_V3V5** | **92.15** | **LOW** | **84.6** | **84.6** | **Stool** | **0.07** |
| **WGACA01T1F15228** | **otu_647_V1V3** | **95.14** | **LOW** | **78.47** | **79.5** | **Stool** | **0.19** |
| **WGACA01T1F15325** | **otu_774_V3V5** | **91.53** | **LOW** | **85.79** | **85.98** | **Stool** | **0.14** |
| **WGACA01T1F15141** | **otu_1233_V3V5** | **92.11** | **LOW** | **84.6** | **84.6** | **Stool** | **0.07** |
| **WGACA01T1F15334** | **otu_774_V3V5** | **93.05** | **LOW** | **85.79** | **85.98** | **Stool** | **0.14** |
| **WGACA10T1R14970** | **otu_422_V1V3** | **63.77** | **LOW** | **89.11** | **89.11** | **Stool** | **0.02** |
| **WGACA01T1F40204** | **otu_766_V3V5** | **97.14** | **LOW** | **87.59** | **87.78** | **Stool** | **0.17** |
| **WGACA01T1R40230** | **otu_774_V3V5** | **97.98** | **LOW** | **85.79** | **85.98** | **Stool** | **0.14** |
| **WGACA01T1R40215** | **otu_422_V1V3** | **61.73** | **LOW** | **89.11** | **89.11** | **Stool** | **0.02** |
| **WGACA01T1F40335** | **otu_769_V1V3** | **95** | **LOW** | **73.81** | **73.65** | **Stool** | **0.02** |
| **WGACA01T1F40468** | **otu_666_V1V3** | **93.04** | **LOW** | **77.34** | **77.34** | **Stool** | **0.15** |
| **WGACA01T1F40474** | **otu_563_V3V5** | **99.44** | **LOW** | **79.7** | **79.7** | **Stool** | **0.13** |
| **WGACA01T1R40611** | **otu_422_V1V3** | **62.02** | **LOW** | **89.11** | **89.11** | **Stool** | **0.02** |
| **WGACA50953** | **otu_766_V3V5** | **96.22** | **LOW** | **87.59** | **87.78** | **Stool** | **0.17** |
| **WGACA50989** | **otu_774_V3V5** | **91.87** | **LOW** | **85.79** | **85.98** | **Stool** | **0.14** |
| **WGACA50994** | **otu_1233_V3V5** | **92.95** | **LOW** | **84.6** | **84.6** | **Stool** | **0.07** |
| **WGACA51383** | **otu_563_V3V5** | **99.63** | **LOW** | **79.7** | **79.7** | **Stool** | **0.13** |
| **WGACA51384** | **otu_774_V3V5** | **93.05** | **LOW** | **85.79** | **85.98** | **Stool** | **0.14** |
| **WGACA51476** | **otu_563_V3V5** | **99.63** | **LOW** | **79.7** | **79.7** | **Stool** | **0.13** |
| **WGACA51541** | **otu_1233_V3V5** | **96.83** | **LOW** | **84.6** | **84.6** | **Stool** | **0.07** |
| **WGACA51548** | **otu_1162_V3V5** | **100** | **LOW** | **89.67** | **89.67** | **Stool** | **0.15** |
| **WGACA51566** | **otu_624_V3V5** | **95.2** | **LOW** | **86.63** | **86.83** | **Stool** | **0.11** |
| **WGACA51821** | **otu_1233_V3V5** | **96.83** | **LOW** | **84.6** | **84.6** | **Stool** | **0.07** |
| **WGACA01T1F43701** | **otu_909_V1V3** | **94.17** | **LOW** | **82.67** | **83.72** | **Stool** | **0.15** |
| **WGACA17T1R43532** | **otu_1054_V1V3** | **64.29** | **LOW** | **85.51** | **85.51** | **Saliva** | **0.01** |
| **WGACA01T1F15241** | **otu_930_V1V3** | **94.79** | **LOW** | **93.54** | **93.54** | **Anterior nares** | **0.01** |
| **WGACA01T1R40264** | **otu_1172_V3V5** | **60.61** | **LOW** | **93.5** | **92.93** | **L_Antecubital fossa** | **0.07** |
| **WGACA01T1F40433** | **otu_489_V1V3** | **100** | **LOW** | **93.42** | **93.42** | **R_Antecubital fossa** | **0.19** |
| **WGACA01T1F40408** | **otu_1068_V3V5** | **96.37** | **LOW** | **95.22** | **96.37** | **R_Antecubital fossa** | **0.05** |
| **WGACA01T1F40592** | **otu_1253_V3V5** | **90.93** | **LOW** | **97.23** | **97.23** | **Stool** | **0.03** |
| **WGACA50861** | **otu_1253_V3V5** | **96.21** | **LOW** | **97.23** | **97.23** | **Stool** | **0.03** |
| **WGACA51211** | **otu_848_V1V3** | **91.46** | **LOW** | **92.6** | **92.6** | **L_Antecubital fossa** | **0.17** |
| **WGACA51354** | **otu_1149_V3V5** | **93.87** | **LOW** | **92.57** | **92.57** | **Stool** | **0.18** |
| **WGACA51461** | **otu_1068_V3V5** | **94.06** | **LOW** | **95.22** | **96.37** | **R_Antecubital fossa** | **0.05** |
| **WGACA51575** | **otu_1253_V3V5** | **95.58** | **LOW** | **97.23** | **97.23** | **Stool** | **0.03** |
| **WGACA01T1F43784** | **otu_1284_V1V3** | **94.26** | **LOW** | **95.43** | **97.52** | **Stool** | **0.13** |
| WGACA01T1F15093 | otu_700_V1V3 | 100 | LOW | 100 | 100 | Stool | 0.63 |
| WGACA44T1F15004 | otu_620_V1V3 | 97.8 | LOW | 98.75 | 99.58 | Stool | 0.17 |
| WGACA01T1F15285 | otu_700_V1V3 | 99.7 | LOW | 100 | 100 | Stool | 0.63 |
| WGACA57T1F15017 | otu_620_V1V3 | 97.9 | LOW | 98.75 | 99.58 | Stool | 0.17 |
| WGACA62T1F15022 | otu_90_V3V5 | 98.47 | LOW | 99.04 | 99.04 | Stool | 0.87 |
| WGACA01T1F15340 | otu_914_V3V5 | 81.9 | LOW | 99.23 | 99.23 | Stool | 0.06 |
| WGACA01T1R15102 | otu_84_V1V3 | 66.67 | LOW | 99.57 | 97.19 | Supragingival plaque | 0.78 |
| WGACA01T1R15231 | otu_38_V3V5 | 56.41 | LOW | 98.51 | 95.52 | Buccal mucosa | 0.82 |
| WGACA26T1F39957 | otu_308_V3V5 | 99.35 | LOW | 100 | 100 | Stool | 0.76 |
| WGACA01T1F40150 | otu_1194_V3V5 | 100 | LOW | 100 | 100 | Stool | 0.12 |
| WGACA01T1F40072 | otu_556_V3V5 | 99.41 | LOW | 99.6 | 100 | Stool | 0.61 |
| WGACA01T1F40261 | otu_556_V3V5 | 96.64 | LOW | 99.6 | 100 | Stool | 0.61 |
| WGACA71T1F40002 | otu_99_V3V5 | 99.26 | LOW | 99.26 | 97.24 | Stool | 0.89 |
| WGACA01T1F40282 | otu_99_V3V5 | 99.26 | LOW | 99.26 | 97.24 | Stool | 0.89 |
| WGACA01T1F40194 | otu_1194_V3V5 | 99.24 | LOW | 100 | 100 | Stool | 0.12 |
| WGACA01T1F40102 | otu_33_V3V5 | 99.79 | LOW | 98.8 | 99.6 | Stool | 0.59 |
| WGACA77T1F40008 | otu_350_V3V5 | 100 | LOW | 100 | 100 | Stool | 0.75 |
| WGACA01T1F40114 | otu_422_V3V5 | 100 | LOW | 100 | 100 | Stool | 0.74 |
| WGACA90T1F40021 | otu_321_V1V3 | 94.88 | LOW | 99.79 | 99.79 | Stool | 0.91 |
| WGACA01T1F40312 | otu_99_V3V5 | 99.08 | LOW | 99.26 | 97.24 | Stool | 0.89 |
| WGACA15T1R39946 | otu_350_V3V5 | 100 | LOW | 100 | 100 | Stool | 0.75 |
| WGACA01T1R40258 | otu_350_V3V5 | 100 | LOW | 100 | 100 | Stool | 0.75 |
| WGACA01T1F40326 | otu_395_V3V5 | 95.77 | LOW | 100 | 100 | Stool | 0.78 |
| WGACA01T1F40328 | otu_556_V3V5 | 96.25 | LOW | 99.6 | 100 | Stool | 0.61 |
| WGACA01T1F40424 | otu_398_V3V5 | 99.78 | LOW | 99.78 | 99.78 | Stool | 0.68 |
| WGACA01T1F40331 | otu_321_V1V3 | 100 | LOW | 99.79 | 99.79 | Stool | 0.91 |
| WGACA01T1F40350 | otu_99_V3V5 | 99.08 | LOW | 99.26 | 97.24 | Stool | 0.89 |
| WGACA01T1F40553 | otu_1194_V3V5 | 100 | LOW | 100 | 100 | Stool | 0.12 |
| WGACA01T1F40389 | otu_99_V3V5 | 99.08 | LOW | 99.26 | 97.24 | Stool | 0.89 |
| WGACA01T1F40678 | otu_163_V1V3 | 97.92 | LOW | 99.17 | 99.17 | Stool | 0.92 |
| WGACA01T1F40585 | otu_321_V1V3 | 99.11 | LOW | 99.79 | 99.79 | Stool | 0.91 |
| WGACA01T1R40614 | otu_806_V3V5 | 58.9 | LOW | 99.63 | 95.75 | Saliva | 0.05 |
| WGACA48045 | otu_430_V3V5 | 81.51 | LOW | 100 | 100 | Stool | 0.51 |
| WGACA47860 | otu_395_V3V5 | 100 | LOW | 100 | 100 | Stool | 0.78 |
| WGACA47765 | otu_620_V1V3 | 98.88 | LOW | 98.75 | 99.58 | Stool | 0.17 |
| WGACA48053 | otu_11_V3V5 | 100 | LOW | 100 | 100 | Tongue dorsum | 0.99 |
| WGACA47986 | otu_398_V3V5 | 100 | LOW | 99.78 | 99.78 | Stool | 0.68 |
| WGACA47903 | otu_398_V3V5 | 97.95 | LOW | 99.78 | 99.78 | Stool | 0.68 |
| WGACA47994 | otu_90_V3V5 | 84.87 | LOW | 99.04 | 99.04 | Stool | 0.87 |
| WGACA47825 | otu_1194_V3V5 | 97.39 | LOW | 100 | 100 | Stool | 0.12 |
| WGACA50869 | otu_178_V3V5 | 55.17 | LOW | 99.06 | 94.92 | L_Antecubital fossa | 0.53 |
| WGACA50871 | otu_321_V1V3 | 99.7 | LOW | 99.79 | 99.79 | Stool | 0.91 |
| WGACA50909 | otu_410_V1V3 | 97.34 | LOW | 99.36 | 99.36 | Stool | 0.71 |
| WGACA50917 | otu_321_V1V3 | 99.71 | LOW | 99.79 | 99.79 | Stool | 0.91 |
| WGACA50933 | otu_395_V3V5 | 99.8 | LOW | 100 | 100 | Stool | 0.78 |
| WGACA50936 | otu_142_V1V3 | 98.76 | LOW | 98.92 | 92.47 | Stool | 0.85 |
| WGACA50941 | otu_556_V3V5 | 100 | LOW | 99.6 | 100 | Stool | 0.61 |
| WGACA50949 | otu_556_V3V5 | 99.6 | LOW | 99.6 | 100 | Stool | 0.61 |
| WGACA50951 | otu_99_V3V5 | 98.7 | LOW | 99.26 | 97.24 | Stool | 0.89 |
| WGACA50958 | otu_410_V1V3 | 99.48 | LOW | 99.36 | 99.36 | Stool | 0.71 |
| WGACA50975 | otu_142_V1V3 | 98.76 | LOW | 98.92 | 92.47 | Stool | 0.85 |
| WGACA50977 | otu_556_V3V5 | 96.99 | LOW | 99.6 | 100 | Stool | 0.61 |
| WGACA50982 | otu_90_V3V5 | 84.63 | LOW | 99.04 | 99.04 | Stool | 0.87 |
| WGACA50992 | otu_190_V1V3 | 100 | LOW | 99.51 | 99.51 | Stool | 0.53 |
| WGACA51017 | otu_99_V3V5 | 99.05 | LOW | 99.26 | 97.24 | Stool | 0.89 |
| WGACA51036 | otu_256_V1V3 | 99.7 | LOW | 99.7 | 99.7 | Stool | 0.14 |
| WGACA51039 | otu_321_V1V3 | 99.7 | LOW | 99.79 | 99.79 | Stool | 0.91 |
| WGACA51049 | otu_556_V3V5 | 99.8 | LOW | 99.6 | 100 | Stool | 0.61 |
| WGACA51097 | otu_556_V3V5 | 96.65 | LOW | 99.6 | 100 | Stool | 0.61 |
| WGACA51124 | otu_256_V1V3 | 99.7 | LOW | 99.7 | 99.7 | Stool | 0.14 |
| WGACA51131 | otu_556_V3V5 | 96.67 | LOW | 99.6 | 100 | Stool | 0.61 |
| WGACA51133 | otu_556_V3V5 | 96.55 | LOW | 99.6 | 100 | Stool | 0.61 |
| WGACA51154 | otu_142_V1V3 | 99.39 | LOW | 98.92 | 92.47 | Stool | 0.85 |
| WGACA51166 | otu_410_V1V3 | 100 | LOW | 99.36 | 99.36 | Stool | 0.71 |
| WGACA51231 | otu_200_V1V3 | 97.75 | LOW | 97.49 | 98.33 | Stool | 0.94 |
| WGACA51232 | otu_608_V3V5 | 97.52 | LOW | 98.47 | 98.47 | Stool | 0.41 |
| WGACA51235 | otu_914_V3V5 | 81.9 | LOW | 99.23 | 99.23 | Stool | 0.06 |
| WGACA51325 | otu_292_V3V5 | 100 | LOW | 100 | 79.04 | Stool | 0.36 |
| WGACA51309 | otu_133_V3V5 | 99.81 | LOW | 98.44 | 99.81 | Stool | 0.65 |
| WGACA51336 | otu_21_V3V5 | 100 | LOW | 100 | 100 | Supragingival plaque | 1 |
| WGACA51380 | otu_163_V1V3 | 98.53 | LOW | 99.17 | 99.17 | Stool | 0.92 |
| WGACA51394 | otu_133_V3V5 | 99.8 | LOW | 98.44 | 99.81 | Stool | 0.65 |
| WGACA51412 | otu_99_V3V5 | 97.79 | LOW | 99.26 | 97.24 | Stool | 0.89 |
| WGACA51430 | otu_608_V3V5 | 96.95 | LOW | 98.47 | 98.47 | Stool | 0.41 |
| WGACA51433 | otu_133_V3V5 | 99.78 | LOW | 98.44 | 99.81 | Stool | 0.65 |
| WGACA51436 | otu_7_V3V5 | 84.29 | LOW | 100 | 100 | Supragingival plaque | 1 |
| WGACA51456 | otu_682_V1V3 | 99.73 | LOW | 100 | 100 | Stool | 0.13 |
| WGACA51512 | otu_108_V3V5 | 98.32 | LOW | 99.81 | 99.43 | Anterior nares | 0.6 |
| WGACA51582 | otu_1047_V3V5 | 97.55 | LOW | 98.11 | 98.11 | Stool | 0.37 |
| WGACA51612 | otu_133_V3V5 | 99.6 | LOW | 98.44 | 99.81 | Stool | 0.65 |
| WGACA51676 | otu_430_V3V5 | 86.76 | LOW | 100 | 100 | Stool | 0.51 |
| WGACA51632 | otu_74_V1V3 | 99.41 | LOW | 98.92 | 99.78 | Stool | 0.94 |
| WGACA51736 | otu_90_V3V5 | 84.53 | LOW | 99.04 | 99.04 | Stool | 0.87 |
| WGACA51778 | otu_99_V3V5 | 97.79 | LOW | 99.26 | 97.24 | Stool | 0.89 |
| WGACA51787 | otu_120_V3V5 | 96.94 | LOW | 98.7 | 97.95 | Subgingival plaque | 0.98 |
| WGACA51803 | otu_174_V3V5 | 95.02 | LOW | 99.43 | 93.87 | R_Antecubital fossa | 0.17 |
| WGACA51823 | otu_551_V3V5 | 99.28 | LOW | 100 | 100 | Stool | 0.09 |
| WGACA51831 | otu_174_V3V5 | 93.56 | LOW | 99.43 | 93.87 | R_Antecubital fossa | 0.17 |
| WGACA51964 | otu_133_V3V5 | 99.39 | LOW | 98.44 | 99.81 | Stool | 0.65 |
| WGACA52002 | otu_430_V3V5 | 100 | LOW | 100 | 100 | Stool | 0.51 |
| WGACA01T1F43804 | otu_142_V1V3 | 98.4 | LOW | 98.92 | 92.47 | Stool | 0.85 |
| WGACA01T1F43734 | otu_142_V1V3 | 99.52 | LOW | 98.92 | 92.47 | Stool | 0.85 |
| WGACA59T1F43574 | otu_51_V3V5 | 98.74 | LOW | 99.82 | 99.82 | Vaginal introitus | 0.47 |
| WGACA62T1F43577 | otu_142_V1V3 | 99.3 | LOW | 98.92 | 92.47 | Stool | 0.85 |
| WGACA01T1F43678 | otu_321_V1V3 | 99.06 | LOW | 99.79 | 99.79 | Stool | 0.91 |
| WGACA70T1F43585 | otu_181_V1V3 | 78.77 | LOW | 99.4 | 90.24 | L_Antecubital fossa | 0.16 |
| WGACA01T1F43865 | otu_142_V1V3 | 98.84 | LOW | 98.92 | 92.47 | Stool | 0.85 |
| WGACA01T1F43867 | otu_200_V1V3 | 99.25 | LOW | 97.49 | 98.33 | Stool | 0.94 |
| WGACA01T1F15065 | otu_1057_V3V5 | 99.04 | CHIMERIC | 96.49 | 93.19 | Stool | 0.31 |
| WGACA01T1F15306 | otu_1046_V1V3 | 74.24 | CHIMERIC | 94.42 | 94.64 | Stool | 0.36 |
| WGACA37T1R14997 | otu_294_V3V5 | 99.21 | CHIMERIC | 97.52 | 97.52 | Stool | 0.82 |
| WGACA01T1R15201 | otu_954_V3V5 | 100 | CHIMERIC | 91.48 | 91.48 | Stool | 0.37 |
| WGACA22T1F39953 | otu_502_V3V5 | 97.83 | CHIMERIC | 99.21 | 99.21 | Stool | 0.62 |
| WGACA01T1F40238 | otu_784_V3V5 | 91.6 | CHIMERIC | 95.32 | 95.32 | Anterior nares | 0.02 |
| WGACA78T1F40009 | otu_1205_V3V5 | 94.4 | CHIMERIC | 97.32 | 97.51 | Stool | 0.13 |
| WGACA01T1F40119 | otu_195_V3V5 | 97.78 | CHIMERIC | 98.39 | 98.39 | Stool | 0.95 |
| WGACA50T1R39981 | otu_1353_V1V3 | 59.3 | CHIMERIC | 94.01 | 94.01 | Stool | 0.28 |
| WGACA01T1F40383 | otu_1205_V3V5 | 94.69 | CHIMERIC | 97.32 | 97.51 | Stool | 0.13 |
| WGACA50948 | otu_1163_V3V5 | 100 | CHIMERIC | 97.65 | 97.65 | Stool | 0.29 |
| WGACA50965 | otu_1338_V1V3 | 82.03 | CHIMERIC | 96.52 | 96.31 | Stool | 0.26 |
| WGACA51019 | otu_775_V3V5 | 99.52 | CHIMERIC | 98.89 | 99.08 | Stool | 0.48 |
| WGACA51090 | otu_1057_V3V5 | 99.76 | CHIMERIC | 96.49 | 93.19 | Stool | 0.31 |
| WGACA51703 | otu_502_V3V5 | 99.21 | CHIMERIC | 99.21 | 99.21 | Stool | 0.62 |
| WGACA51713 | otu_849_V3V5 | 79.51 | CHIMERIC | 96.18 | 96.18 | Stool | 0.39 |
| WGACA51800 | otu_502_V3V5 | 99.01 | CHIMERIC | 99.21 | 99.21 | Stool | 0.62 |
| WGACA51880 | otu_502_V3V5 | 98.82 | CHIMERIC | 99.21 | 99.21 | Stool | 0.62 |
| WGACA51941 | otu_502_V3V5 | 99.21 | CHIMERIC | 99.21 | 99.21 | Stool | 0.62 |
| WGACA51952 | otu_502_V3V5 | 99.21 | CHIMERIC | 99.21 | 99.21 | Stool | 0.62 |
| WGACA01T1F43834 | otu_701_V1V3 | 97.83 | CHIMERIC | 96.95 | 96.95 | Stool | 0.61 |
| WGACA01T1F43801 | otu_1083_V3V5 | 88.74 | CHIMERIC | 96.79 | 96.79 | Tongue dorsum | 0.28 |
