# Supplementary material for: The “Most Wanted” Taxa from the Human Microbiome for Whole Genome Sequencing
Source: PLoS One. 2012 Jul 26;7(7):e41294. doi: 10.1371/journal.pone.0041294 (PMC3406062; doi:10.1371/journal.pone.0041294)
Supplement: Document S1 — Exploring high chimera rates among HMP OTUs. (DOCX) [file pone.0041294.s008.docx]

**Supplemental Document 1. Exploring high chimera rates among HMP OTUs**

There were a large number of chimeras in the AbundantOTU consensus sequences; 46.3% of the V1-V3 and 44.8% of the V3-V5 consensus sequences, representing 5.6% and 7.2% of total reads, respectively, were flagged as chimeric using either UCHIME with the GOLD reference database (UCHIME ref) or UCHIME de novo (in which the number of sequence reads in each OTU was used for the UCHIME “size” parameter). A similar percentage of reads from these two data sets were also identified as chimeric using the ChimeraSlayer implementation in the Mothur package of tools. Other 16S datasets, using similar primers and put through a similar pipeline, have not displayed such high rates of chimerism (Fodor lab, unpublished data). Since it was unclear why the HMP data set suffered such a high rate of chimerism, we examined these sequences further.

V1-V3 and V3-V5 16S sequences were not filtered for chimeras prior to clustering with AbundantOTU. To verify that AbundantOTU was not artificially inflating chimeras by creating chimeric consensus sequences out of non-chimeric reads, we ran all *23,515,839* V1-V3 and *29,567,447* V3-V5 454 16S sequences through UCHIME with the GOLD database as a reference. In general, there was good agreement between the number of reads called chimeric by UCHIME within an HMP OTU and the UCHIME classification of its consensus sequence (Supplemental Fig. 1). For V1-V3 (Supplemental Fig. 1 left panel), there were 754 OTUs that had <=10% of their reads flagged as chimeric. Of these, only five had consensus sequences that were called chimeric. Of the 867 V1-V3 OTUs that had <=50% of their reads flagged as chimeric, only 28 had consensus sequences called chimeric. Put another way, of the 578 OTUs in V1-V3 called chimeric by UCHIME, only 4.8% (28 out of 578) had fewer than half of the individual reads called chimeric. In summary, while it is possible that AbundantOTU occasionally produces a chimeric consensus sequence, for >95% of the chimeric V1-V3 consensus sequences, the majority of the reads were also chimeric.

A similar pattern was observed for V3-V5 HMP OTUs (Supplemental Fig. 1; right panel). Of the 513 consensus sequences called chimeric by UCHIME Ref, 485 of them (94.5%) had, at least, half of their reads also classified as chimeric by UCHIME ref. We concluded that, for the vast majority of consensus sequences, chimeras were not introduced by AbundantOTU.

**UCHIME ref (to GOLD database) and UCHIME de novo are generally in broad agreement.**

We used UCHIME both in de novo mode and in reference mode to characterize HMP OTUs (Supplemental Fig. 2). Relatively modest numbers of chimeras were added by considering both methods when compared to either method alone: for example, there are 527 V1-V3 HMP OTUs called by both methods, 89 called only by UCHIME de novo and 51 called just by UCHIME ref. For this study, however, we favored a conservative approach and considered an OTU chimeric if UCHIME flagged it as chimeric by either method.

**Alignment against the Silva database suggests that UCHIME’s default threshold may be too high (i.e. non-chimeric sequences are being called chimeric).**

For both V1-V3 and V3-V5 HMP OTU’s, as the chimera score assigned by UCHIME increased, the percent identity to Silva decreased (Supplemental Fig. 3). There were, however, a number of chimeras called chimeric by either UCHIME de novo or UCHIME ref or both that had >97% identity in the Silva database (Supplemental Fig. 3). This raises the possibility that UCHIME’s default cutoff may be too harsh; that is, UCHIME is calling sequences chimeric that are either not chimeric or have only a modest degree of chimerism at the ends of the consensus sequence. Again, for this study, we took a conservative approach and regarded any sequence flagged as chimeric by UCHIME, using default parameters, chimeric.
