# Supplementary material for: The “Most Wanted” Taxa from the Human Microbiome for Whole Genome Sequencing
Source: PLoS One. 2012 Jul 26;7(7):e41294. doi: 10.1371/journal.pone.0041294 (PMC3406062; doi:10.1371/journal.pone.0041294)
Supplement: Document S2 — Determining percent identity prioritization thresholds. (DOCX) [file pone.0041294.s009.docx]

**Supplemental Document 2. Determining Percent Identity Prioritization Thresholds**

In order to determine which microbes should be prioritized for whole-genome sequencing, we sought a rational percent identity cutoff as a definition of a “novel” sequence. Toward that end, we asked what percent identity was associated with sequences being assigned different taxonomic names.

For each HMP OTU sharing 100% sequence identity to a ‘named’ cultured organism (Fig. 3E), we compared the genus- and species-level taxonomic assignment given to that cultured organism to the genus- and species-level taxonomic assignment given to the best match from the GOLD database (where the GOLD sequence identities ranged from 83%-100%). We compared 262 genus names and 188 species names, leaving out organisms with identical identifiers or, for the latter comparison, organisms with only a *sp.* species designation. The percent of matches for each GOLD identity range was calculated (Supplemental Table 2). While there was never perfect agreement between names in any GOLD identity range, except for the very few OTUs falling within the 98-98.9% identity window, we observed much less ‘species’ agreement when GOLD sequences shared <98% ID to the HMP OTU. Furthermore, there was no agreement between species names when the GOLD identity was below 97%. The drop in genus-level concordance was not as dramatic, but we did observe an appreciable decrease below 98% identity. However, unlike the species comparisons, the number of genus-level concordance never went to zero, even when the sequence identity between GOLD and the HMP OTU was less than 90%.

Though our assessment was very limited in scope and subject to imprecise and uneven taxonomic assignments across bacterial taxa [33], we used this information to draw two lines in our analysis, one at 98% and another at 90%, to create three priority groups, “low”, “medium” and “high”, to prioritize HMP OTUs for isolation and sequencing of the organisms that they represent (Figure 2, Table 2).
